# Infection may play an important role in the pathogenesis of alveolar osteonecrosis following facial herpes zoster: a case report and literature review

**DOI:** 10.1186/s12903-024-04202-z

**Published:** 2024-04-02

**Authors:** Kaikai Huang, Youyuan Wang, Yuhua Huang, Shanshan Han, Yu Yang, Pinghua Qu, Baoying Liang, Qingyu Zhen, Wenting Chen, Ying Lin

**Affiliations:** 1 The Second Affiliated Hospital of Guangzhou University of Chinese Medicine, Guangzhou, 510000 China; 2 Department of Oral and Maxillofacial Surgery, Sun Yat-sen Memorial Hospital, Sun Yat-sen University, Guangzhou, 510000 China; 3 The Second Clinical School of Guangzhou University of Chinese Medicine, Guangzhou, 510000 China

**Keywords:** Herpes zoster, Tooth exfoliation, Alveolar osteonecrosis, Infection, Complication

## Abstract

**Background:**

Herpes zoster (HZ) is one of the most common skin diseases caused by viruses. Facial HZ develops when the *varicella-zoster virus* affects the trigeminal nerve, and alveolar osteonecrosis is a rare complication. However, the exact pathogenesis of postherpetic alveolar osteonecrosis remains unclear.

**Case description:**

We encountered a patient who presented to the dermatology clinic with facial HZ and tooth exfoliation in the upper right jaw, and panoramic radiography revealed decreased bone density and poor alveolar socket healing in his right maxilla. Biopsy of the alveolar process revealed fragments of nonvital lamellar bone, which were devoid of osteoblasts and osteocytes and were surrounded by numerous neutrophils and bacterial aggregates. Thus, the diagnosis of alveolar osteonecrosis following facial HZ was confirmed. He then underwent resection of the osteonecrotic tissue. The pathological findings of postoperative tissue were similar to those of previous biopsies. *Varicella-zoster virus* and multiple types of bacteria were detected through next-generation sequencing, and the species of bacteria were consistent with the results of bacterial culture. Antibiotics and valaciclovir were administered during the perioperative period. The patient showed good recovery at the 9-month follow-up.

**Conclusions:**

The coexistence of bacterial and viral infection may play an important role in the pathogenesis of alveolar osteonecrosis following HZ. To our knowledge, we are the first to directly explore microbial pathogens in a case of postherpetic alveolar osteonecrosis through next-generation sequencing and bacterial culture. We recommend that oral examinations be carefully conducted for patients who are diagnosed with facial HZ, even if their facial rashes have faded away. We suggest that a prolonged and full-dose antiviral therapy course may be beneficial for the treatment of facial HZ with intraoral lesions. The implementation of dental preventive measures should be considered for patients with facial HZ. The application of antibiotics and excision of necrotic bone may reduce the abundance of bacteria in lesions and improve wound healing.

## Background

Herpes zoster (HZ) is one of the most common skin diseases caused by viruses, and up to one-third of humans may be affected during their lives [1]. Facial HZ develops when *varicella-zoster virus* (VZV) affects the trigeminal nerve [2]. Herpetic neuralgia and Ramsay Hunt syndrome are well-known complications of facial HZ [2, 3]. Another rare, severe complication is alveolar osteonecrosis, which can be easily overlooked, as it may occur long after the onset of HZ [4]. Only 46 such cases had been reported as of 2014 [4].

Alveolar osteonecrosis is a severe bone disease (osteonecrosis) that affects the jaws (the maxilla and the mandible). The definitive diagnosis of alveolar osteonecrosis depends on the pathological characteristics of osteonecrosis. Alveolar osteonecrosis is usually considered related to certain kinds of drugs (medication-related osteonecrosis of the jaws (MRONJ) due to antiangiogenic agents or antiresorptive drugs such as bisphosphonates and denosumab), radiotherapy (osteoradionecrosis), bacterial infection (osteomyelitis) and metastatic jaw disease [5–7]. However, the exact pathogenesis of postherpetic alveolar osteonecrosis remains unclear.

Herein, we report the case of a patient with HZ and ipsilateral tooth exfoliation who was later diagnosed with alveolar osteonecrosis. We demonstrate the important role of infection in the pathogenesis of alveolar osteonecrosis through pathological characteristics, next-generation sequencing (NGS) and bacterial culture.

## Case presentation

A 67-year-old man presented to the dermatology clinic of the Second Affiliated Hospital of Guangzhou University of Chinese Medicine with a 5-week history of erythema and clustered blisters accompanied by great pain in the right face. He had a severe toothache in the upper right jaw, visited the stomatologist in a local hospital 4 weeks prior and was diagnosed with acute periodontitis and HZ. He was prescribed intravenous ceftizoxime 1 g/d and metronidazole 0.5 g/d, as well as oral acetaminophen for 2 weeks. However, the rashes on his face worsened, and he was subsequently referred to the dermatology clinic in the local hospital. A 10-day regimen of oral valacyclovir 1 g twice a day was initiated. However, the patient’s intense pain was not relieved and four teeth of his upper right jaw exfoliated in succession 10 days before he visited our clinic.

The patient had a 30-year on-and-off history of toothache. He saw the stomatologist and took painkillers at the very beginning. Then, he took metamizole sodium and phenylbutazone tablets every time the toothache attacked, and he hardly went to the hospital to receive standardized treatment, even when he lost several molar teeth many years earlier. The patient also had a history of hypertension and infection with *hepatitis B virus* (HBV) for years. He had no previous history of tumors, local radiotherapy or other therapy with antiangiogenic agents or antiresorptive drugs such as bisphosphonates and denosumab.

**Fig. 1:**
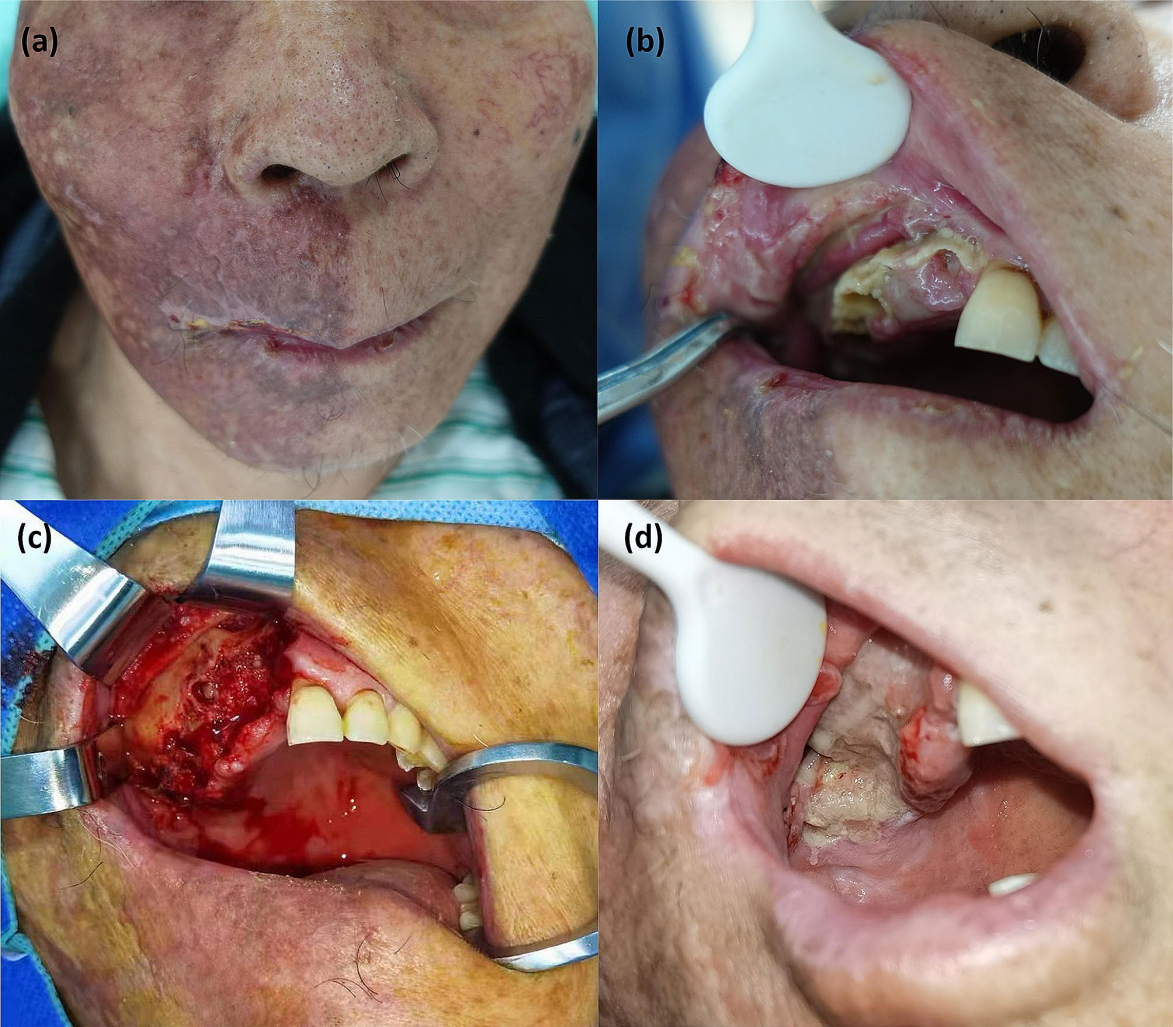
Clinical findings. (**a**) Pigmentation and scars on the right face after herpes zoster. (**b**) Tooth exfoliation, gingival recession, and exposure of the alveolar process in the first quadrant of the maxillary arch extending from tooth 11 to 14. (**c**) Extensive osteonecrosis was excised during debridement. (**d**) Granulation tissue formation was observed three weeks after debridement

Extraoral examination revealed pigmentation and scars on the right half of the face (Fig. 1a). On intraoral examination, it was found that there was a complete loss of crowns from teeth 11 to 17 and 35; there was also some tooth decay, gingival recession, and exposure of the alveolar process in the first quadrant of the maxillary arch extending from teeth 11 to 14 (Fig. 1b). Residual roots of teeth 14, 36 and 48 were also observed.

**Fig. 2:**
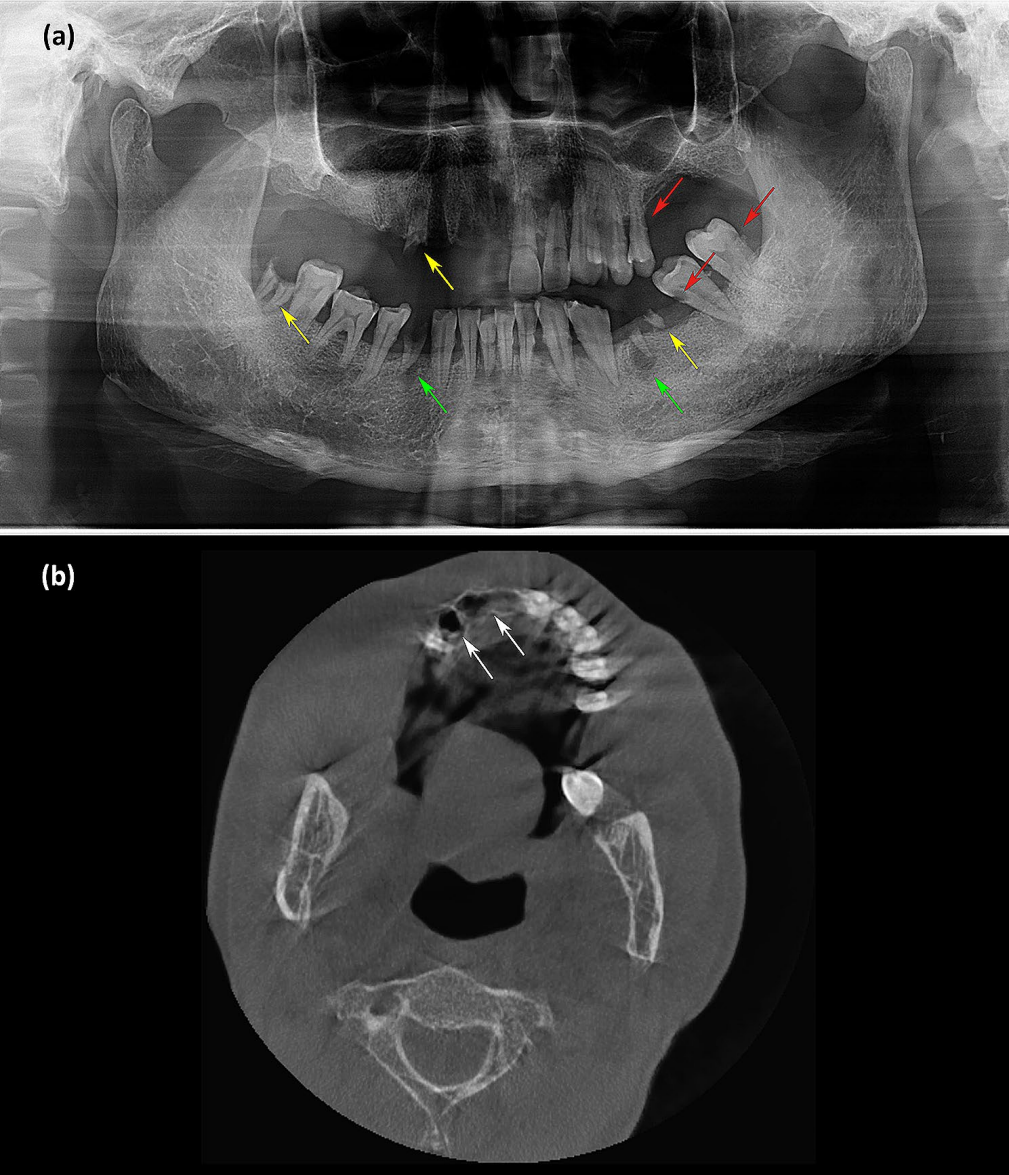
Imaging manifestations. (**a**) Panoramic radiography showed decreased bone density and poor alveolar socket healing in the right maxilla. Decayed teeth (red arrowhead), residual roots of teeth (yellow arrowhead) and periapical cysts (green arrowhead) were found. (**b**) Computed tomography examination revealed empty tooth sockets (white arrowhead) on the right side of the maxilla

Laboratory tests yielded the following results: normal coagulation function, blood glucose, routine urine tests, routine stool tests, electrocardiography and chest radiography. The screening result for *human immunodeficiency virus* (HIV) antibody was negative. Quantitative analysis of HBV DNA yielded a value of 4.18 × 10^5^ IU/mL. Routine blood examination revealed an elevated white blood cell count of 10.84 × 10^9^/L. CRP was 20.8 mg/L. ALT and serum creatinine were slightly elevated at 54 U/L and 138 µmol/L, respectively. Color ultrasonography showed multiple hepatic cysts and renal cysts. Panoramic radiography was conducted 10 days after tooth exfoliation and revealed decreased bone density and poor alveolar socket healing in his right maxilla. Decayed teeth, residual roots of teeth and periapical cysts were found (Fig. 2a). Computed tomography examination revealed empty tooth sockets on the right side of the maxilla (Fig. 2b).

**Fig. 3:**
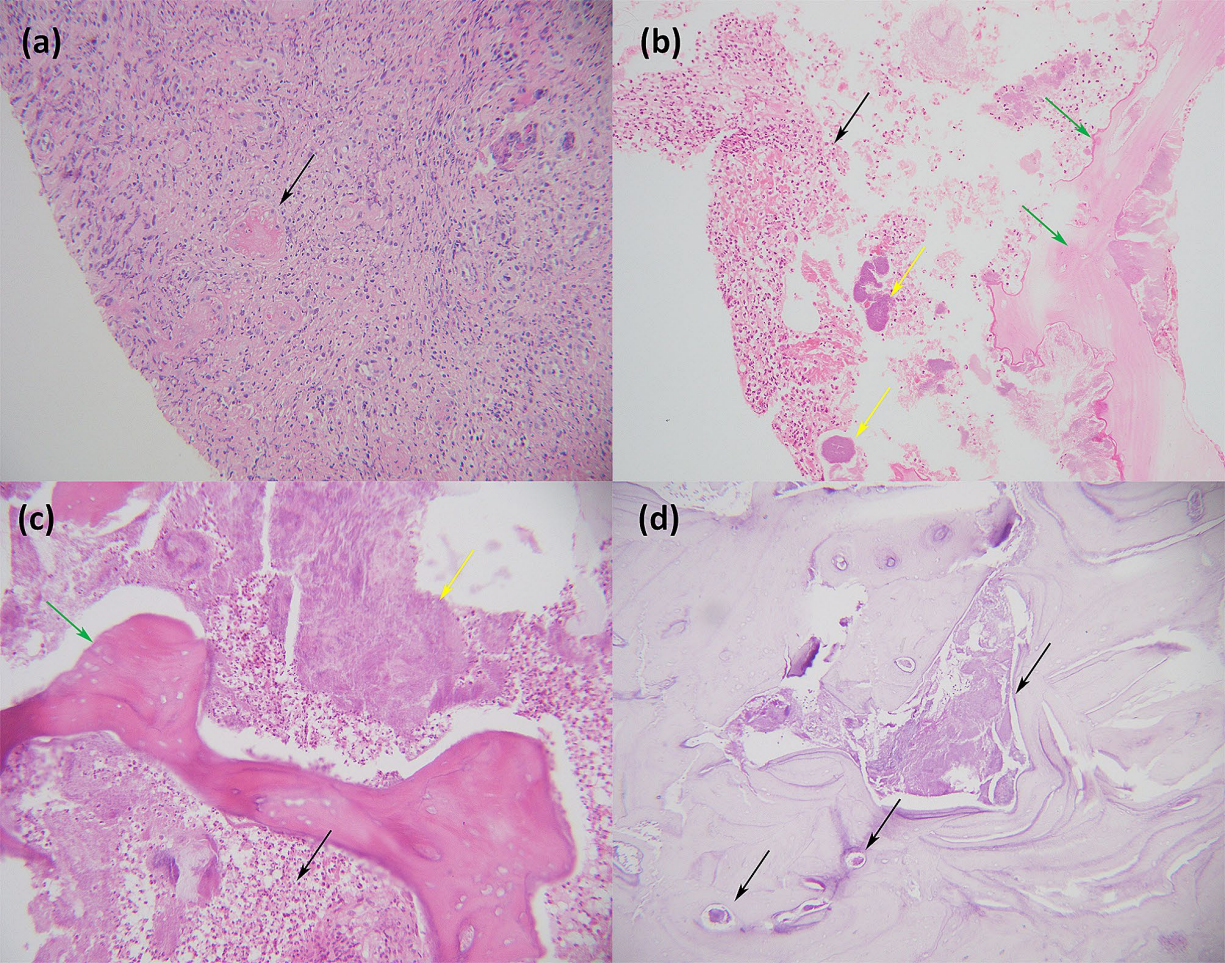
Histological findings. (**a**) The biopsy of the mucous membrane adjacent to the alveolar process revealed infiltration of histiocytes and neutrophils beneath the mucosa. The arrowhead indicates lumen occlusion (Haematoxylin and Eosin ×200). (**b**) The biopsy of the alveolar process showed fragments of nonvital lamellar bone (green arrowhead), which were devoid of osteoblasts and osteocytes and were surrounded by numerous neutrophils (black arrowhead) and bacterial aggregates (yellow arrowhead) (Haematoxylin and Eosin ×200). (**c**) The pathological findings of postoperative tissue were similar to those of previous biopsies, and osteonecrosis (green arrowhead), numerous neutrophils (black arrowhead) and bacterial aggregates (yellow arrowhead) were observed (Haematoxylin and Eosin ×200). (**d**) Arrowheads indicate bacterial aggregates inside the marrow cavity (Periodic Acid-Schiff staining ×100)

Biopsy was conducted from a piece of alveolar process and adjacent mucous membrane. Hyperplasia of the squamous epithelium with no atypia was observed in the oral mucosa. Fibrinoid necrosis of some vascular walls, lumen occlusion, and infiltration of histiocytes and neutrophils were found beneath the mucosa (Fig. 3a). There were also some fragments of nonvital lamellar bone, which were devoid of osteoblasts and osteocytes and were surrounded by numerous neutrophils and bacterial aggregates (Fig. 3b). Thus, the diagnosis of alveolar osteonecrosis following facial HZ was confirmed.

The patient was then transferred to the Department of Oral and Maxillofacial Surgery in Sun Yat-sen Memorial Hospital for resection of the osteonecrotic tissue. Extensive malodorous osteonecrosis was observed in the right maxilla during debridement (Fig. 1c). The pathological findings of postoperative tissue were similar to those of previous biopsies (Fig. 3c). Bacterial aggregates could be seen inside the marrow cavity by Periodic Acid-Schiff staining (Fig. 3d).

A necrotic bone tissue sample was taken for bacterial culture under aerobic and anaerobic conditions. *Prevotella denticola*, *Streptococcus intermedius*, *Actinomycetes oris*, and *Actinomyces viscosus* were then confirmed by matrix-assisted laser desorption/ionization time-of-flight mass spectrometry. Moreover, NGS was conducted from a piece of alveolar process and adjacent mucous membrane. Following DNA extraction, DNA libraries were constructed and sequenced by the MGISEQ-2000 platform [8]. High-quality sequencing data were generated by removing low-quality reads, followed by computational subtraction of human host sequences mapped to the human reference genome (hg19) using Burrows‒Wheeler Alignment [9]. The remaining data obtained by removal of low-complexity reads were classified by simultaneous alignment to the Pathogens Metagenomics Database (PMDB), consisting of bacteria, fungi, viruses and parasites. Finally, *Prevotella, Streptococcus, Lactobacillus, Veillonella, Actinomyces, Candida*, VZV, HBV, *human gammaherpesvirus 4* and *Torque teno virus* (TTV) were detected by NGS. Thus, the coexistence of bacterial and viral infection was confirmed.

During the perioperative period, a 7-day regimen of antiviral treatment (oral valacyclovir 1 g twice a day) and antibiotic therapy (intravenous cefathiamidine 2 g twice a day for 3 days and oral cefuroxime 0.25 g twice a day for 4 days successively) were administered. Granulation tissue formation was observed on the surface of the alveolar wound three weeks after debridement (Fig. 1d). At a follow-up 9 months later, no further tooth exfoliation was found.

## Discussion and conclusions

Although there was no apparent osteonecrosis of the jaw according to panoramic radiographs or CT scans, biopsy of the alveolar process revealed the typical pathological characteristics of osteonecrosis. Thus, the diagnosis of alveolar osteonecrosis following facial HZ can be confirmed before surgery. Alveolar osteonecrosis may appear 9 to 150 days after the onset of facial HZ [10], and tooth exfoliation is one of its most important clinical manifestations [11, 12]. This phenomenon could hardly be explained by coincidence, as reported cases of tooth exfoliation have always occurred on the same side as facial HZ [4]. There must be some underlying factors associated with facial HZ with alveolar osteonecrosis.

MRONJ and osteoradionecrosis could be ruled out in the present case, as the patient did not have a related history. Histological findings did not support metastatic jaw diseases. Some scholars believe that local vasculitis caused by viruses [13], vasoconstriction through sympathetic innervation [14], mechanical compression of the alveolar artery by the swollen alveolar nerve [10], or a hypercoagulable state may be involved in the pathogenesis of postherpetic alveolar osteonecrosis. In our case, the pathological findings of fibrinoid necrosis on the vascular wall and lumen occlusion may support the hypothesis that vascular factors also partially contributed to postherpetic alveolar osteonecrosis.

Notably, the patient had a long-term history of toothache but did not receive standard treatment. Decayed teeth, residual roots of teeth and periapical cysts indicated poor oral hygiene and chronic oral diseases with possible bacterial colonization of the patient. Four teeth of his upper right jaw exfoliated successively after the onset of ipsilateral facial HZ, and interestingly, the adjacent teeth of the upper left jaw seemed not to be affected. An immunosuppressive state, absence of early standardized antiviral treatment, underlying diseases such as tumors, tuberculosis, HIV or HBV infection, and advanced age are considered risk factors for alveolar osteonecrosis in patients with facial HZ involving the maxillary and/or mandibular branch of the trigeminal nerve [11, 12, 14, 15]. Ipsilateral lesions on the buccal mucosa, labial mucosa, tongue, alveolar ridge and soft palate can be affected in facial HZ cases [16]. A subsequent serious bacterial infection, such as septicemia, may occur following HZ [17]. It has been reported that existing periodontitis or pulpitis may lead to more severe alveolar osteonecrosis [9]. Thus, it is reasonable to infer that VZV infection may lead to severe damage to the oral mucosa, which aggravates chronic oral diseases and facilitates bacterial infection.

In view of the lack of in-depth discussion about infection in the previous literature, we attempted to apply comprehensive techniques to analyze the pathogens of postherpetic alveolar osteonecrosis, including bacterial culture, NGS sequencing and histopathological examination. To our knowledge, we are the first to directly explore microbial pathogens in cases of postherpetic alveolar osteonecrosis through NGS sequencing and bacterial culture. The poor oral hygiene, bacterial aggregates observed in the bone marrow cavity of the necrotic bone upon histopathological examination, VZV and multiple types of bacteria detected through NGS sequencing which were consistent with the results of bacterial culture, strongly indicated that the coexistence of bacterial and viral infection may play an important role in the pathogenesis of alveolar osteonecrosis following HZ.

On the other hand, chronic oral diseases may lead to localized immunosuppression, which possibly increases the risk of VZV reactivation. The role of local factors in the outbreak of HZ has been discussed in some studies. It has been reported that HZ can occur in affected sites after local radiotherapy, intra-articular corticosteroid injection and surgical operations [18–21]. The risk of developing HZ in breast cancer patients who have received postoperative radiotherapy may be 3- to 5-fold higher than the incidence in the general population [21]. Obviously, the patient in this case had chronic oral diseases before HZ onset. However, whether preexisting chronic oral diseases may increase the risk of developing HZ remains to be verified by studies on a large sample of patients.

Besides VZV, we should notice that some other viruses such as HBV, *human gammaherpesvirus 4* and TTV were also detected by NGS. High levels of HBV have been confirmed in the blood by quantitative analysis as the case description above. HBV can cause hepatitis, fibrosis, cirrhosis, hepatocellular carcinoma and liver failure [22]. *Human gammaherpesvirus 4*, also known as *Epstein-Barr virus* (EBV), infects more than 95% of the world’s population and is associated with some kinds of lymphoma, nasopharyngeal carcinoma and infectious mononucleosis [23]. EBV establishes a life-long persistence in the human host by infecting B cells, and the cycling of latency and reactivation is ongoing in all infected individuals [23]. TTV DNAemia is universal among the global population and there is now a widespread consensus that TTV should be considered a commensal because no evidence supports a causal association with any human disease [24]. It was unavoidable that the local tissue taken for NGS examination in our case would contain a small amount of blood. Thus, it was reasonable to infer that the HBV, EBV and TTV we detected by NGS originated from the blood. To our knowledge, there are currently no studies reporting the pathogenesis of HBV, EBV and TTV in alveolar osteonecrosis. On the other hand, VZV establishes latency in the cell bodies of axons after primary infection [25]. When reactivated, VZV travels within the axon in anterograde manner to reach the innervated skin and mucous membrane where it causes HZ, characterized by a localized painful vesicular rash [25]. Thus, only when VZV is reactivated can it be detected in local tissue. In addition, postherpetic alveolar osteonecrosis always occurs at the same innervated part of HZ [4]. Therefore, it is reasonable to infer that the VZV we detected in local tissue by NGS is involved in the pathogenesis of postherpetic alveolar osteonecrosis.

Based on the pathogenesis of the disease we discussed above, we recommend that oral examinations be carefully conducted for patients who are diagnosed with facial HZ, even if their facial rashes have faded away. In particular, patients may be first seen in the dermatology department, and they should be referred to the stomatology department for consultation and evaluation.

Early antiviral treatment is important for HZ. The course of antiviral treatment is usually 7 days [26]. In patients who continue to develop new vesicles or who have cutaneous, ocular, neurologic, or motor complications after 7 days of antiviral therapy, extending the duration of antiviral therapy for more than 7 days is recommended [26]. However, there is not yet a guideline regarding the recommended antiviral therapy course for the treatment of facial HZ with intraoral lesions. The NGS results of the abovementioned patient demonstrated the existence of VZV in oral lesions at 5 weeks after the onset of HZ. Thus, we suggest that a prolonged and full-dose antiviral therapy course may be beneficial for the treatment of facial HZ with intraoral lesions, especially when the intraoral mucosa has not recovered after a conventional 7-day therapy course.

In considering prevention, we can refer to MRONJ, as bacterial infection (mainly with actinomycetes) is believed to play an important role in MRONJ [12, 27]. The implementation of dental preventive measures in solid tumor patients with bone metastases treated with bisphosphonates may help to decrease the occurrence of MRONJ from 3.2 to 1.3% [28]. We should also strengthen oral health education and nursing practices regarding facial HZ. The application of early antiviral treatment and antibiotics and the excision of necrotic bone would help to improve wound healing to the greatest extent [29]. The bacteria most frequently associated with MRONJ are *Streptococcus species (spp.), Prevotella spp., Actinomyces spp., Veillonella spp., and Parvimonas micra* [30]. The bacteria are most susceptible to the cephalosporins cefotaxime, cefuroxime and β-lactam antibiotics with β-lactamase inhibitors [30]. The pathogenic agents we detected in this case of postherpetic alveolar osteonecrosis were in accordance with MRONJ, indicating that it was reasonable to choose antibiotics for postherpetic alveolar osteonecrosis according to MRONJ.

The removal of necrotic bone may reduce the abundance of bacteria in lesions, especially in deep tissue [30]. In some previous cases of postherpetic alveolar osteonecrosis, the patient underwent more than one operation [2, 31]. Clinicians should attach importance to timely debridement; this was also a key experience in our successful treatment of this patient.

In conclusion, alveolar osteonecrosis is a rare, severe complication of HZ and may occur long after the onset of HZ. Tooth exfoliation is a sign of alveolar osteonecrosis. We have been the first to directly explore the microbial pathogens in a case of postherpetic alveolar osteonecrosis through NGS sequencing and bacterial culture. We suggest that the coexistence of bacterial and viral infection may play an important role in the pathogenesis of alveolar osteonecrosis following HZ.

## Data Availability

All data underlying the findings and outcome are presented as part of the article and no supplementary source data are required.

## Abbreviations

HZ: Herpes zoster
NGS: Next-generation sequencing
HBV: *Hepatitis B virus*
HIV: *Human immunodeficiency virus*
VZV: *Varicella-zoster virus*
EBV: *Epstein-Barr virus*
TTV: *Torque teno virus*
MRONJ: Medication-related osteonecrosis of the jaw
*spp.*: *Species*
